# 1360. Prevalence of Bacteremia in Hospitalized Patients with Skin and Soft Tissue Infections (SSTI)

**DOI:** 10.1093/ofid/ofab466.1552

**Published:** 2021-12-04

**Authors:** Maria J Suarez, Yu Shia Lin

**Affiliations:** Maimonides Medical Center, Brooklyn, New York

## Abstract

**Background:**

Skin and soft tissue infections (SSTI) are common in outpatient and inpatient settings. The prevalence of positive blood cultures (BC) ranges from 2% to 52%. Because of the variations in published data, the exact prevalence of bacteremia in hospitalized patients with SSTI is unknown. Our objective is to determine the prevalence of bacteremia in hospitalized patients with SSTI.

**Methods:**

Retrospective chart review from January 2017 to December 2018. Patients older than 18 years admitted with SSTI who required BC on admission were included. Patients who met the criteria for systemic inflammatory response syndrome (SIRS)/sepsis or severe SSTI, or had an underlying immunodeficiency underwent BC collection. Patients with diabetic foot ulcer, device related SSTI, necrotizing fasciitis, and osteomyelitis were excluded. Patients were divided into 3 groups: true positive (TP) defined as a true pathogen, false positive (FP) defined as a contaminant, and true negative (TN) defined as no growth in BC. Physician assessment, microorganisms isolated, number of positive bottles/culture sets, and timing of growth were reviewed. Patients’ comorbidities, presence of SIRS, laboratory data, duration of antibiotic use, and length of stay (LOS) were compared.

**Results:**

We screened 583 patients and included 541 patients. The mean age was 62 ± 18.4 years, and 60% were male. 47/ 541 (8.6%) had skin abscesses. 57 patients (11%) had positive BC, of whom 32 were TP (6%), and 25 were FP (5%). 89% of patients (484) had TN BC. The organisms isolated are described in Figures 1 and 2. Patients in the FP and TN groups had prior antibiotic use, compared to TP (P< 0.05). The FP group had a longer LOS and duration of antibiotic use compared to the TN group (p< 0.05). 76% of FP had repeated BC. Beta-lactam antibiotics were mostly used, followed by anti-MRSA antibiotics (40%). We did not find risk factors to predict the likelihood of bacteremia. The outcome was not different among the 3 groups.

Figure 1. Microorganisms isolated from blood cultures of patients with SSTI – True pathogens

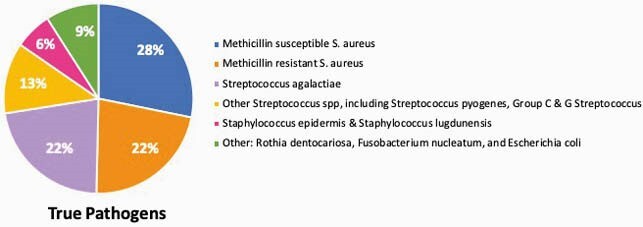

Figure 2. Microorganisms isolated from blood cultures of patients with SSTI – Isolated contaminants

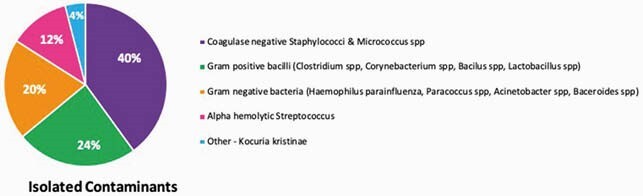

**Conclusion:**

There was a low incidence of true bacteremia (6%) in hospitalized patients with SSTI. More than 90% of TP were predictable causal microorganisms, which are covered by empiric antibiotics. BC may not affect the initial treatment of SSTI. FP BC were associated with an increased LOS, longer antibiotic use, and increased healthcare cost.

**Disclosures:**

**All Authors**: No reported disclosures

